# Impact of a Prototype Combining Recommender Functionality With Structured Documentation on Operator Performance in Calls to Medical Communication Centers: Quasi-Experimental Feasibility Study

**DOI:** 10.2196/87082

**Published:** 2026-05-07

**Authors:** Siri-Linn Schmidt Fotland, Arngeir Berge, Erik Zakariassen, Vivian Midtbø, Valborg Baste, Gro Fonnes, Frode Guribye, Christoph Trattner, Junyong You, Ingrid Hjulstad Johansen

**Affiliations:** 1 National Centre for Emergency Primary Health Care Health and Social Sciences NORCE Research AS Bergen Norway; 2 Department of Global Public Health and Primary Care Faculty of Medicine University of Bergen Bergen Norway; 3 Department of Information Science and Media Studies Faculty of Social Sciences University of Bergen Bergen Norway; 4 Department of Research and Development Norwegian Air Ambulance Foundation Oslo Norway; 5 Digital Systems Enabling Technologies NORCE Research AS Bergen Norway

**Keywords:** telephone triage, telenursing, emergency medical services, after-hours care, decision support systems, clinical, artificial intelligence, recommender systems, nudging

## Abstract

**Background:**

Management of contacts to medical communication centers relies heavily on clinical judgment, contextual understanding, and communication skills. Decision support systems, intended to complement medical expertise, may, due to their rigidity, impede effective caller interaction and may, together with the obligatory documentation of calls, contribute to a workflow that draws attention away from the communication. Recommender systems have demonstrated potential in supporting decision-making across various domains by nudging individuals toward better choices without undermining autonomy. We built a prototype that combined artificial intelligence–based question recommendations with structured documentation (hereafter: the prototype) and conducted a feasibility study to test its influence on operators’ performance.

**Objective:**

This study aimed to examine whether the prototype influenced the operators’ performance during telephone triage. We hypothesized that the prototype would affect medical quality without affecting communication quality.

**Methods:**

A quasi-experimental pre- and posttest feasibility study was conducted in a simulated setting. Twenty-five operators were voluntarily recruited from 5 Norwegian medical communication centers, in which 22 operators contributed to both the pretest (before the prototype) and the posttest (with the prototype). The operators handled the same 15 medical cases presented by simulated callers, with a 5-month interval between the 2 sessions. The question recommender was trained on other data and then fine-tuned on the 15 scenarios used. Audio recordings of the calls were rated using the tool Assessment of Quality in Telephone Triage. Pre- and posttest values were compared, with overall medical and communication quality as the primary outcomes. Secondary outcomes included specific items related to medical content and communication, accuracy of triage, patient safety, call duration, and efficiency.

**Results:**

A total of 320 paired calls were analyzed. Overall medical quality improved significantly with use of the prototype, from a mean of 6.83 points pretest to 7.16 points posttest rated on a 10-point scale (difference 0.34, 95% CI 0.11-0.57; *P*=.004). The effect size was small (Cohen *dz*=0.16). No significant change was observed in overall communication quality, with a mean of 7.06 points pretest and 6.97 points posttest (difference –0.09 points, 95% CI –0.28 to 0.10; *P*=.35). A significant decrease from pre‑ to posttest was observed in the specific items “Collects information about the patient’s location” (*P*<.001) and “Ensures that the triage decision is understandable and feasible” (*P*=.002). None of the remaining secondary outcomes showed significant changes.

**Conclusions:**

The prototype yielded a modest improvement in medical quality within the scenario‑based test environment. Although overall communication quality remained unchanged, aspects of the interaction were negatively affected. Artificial intelligence–based question recommendations combined with structured documentation may serve as useful functionalities within a decision support system, but each functionality requires further testing and development before such technology can be implemented in the triage of unselected, real‑world calls.

## Introduction

Medical communication centers are increasingly being used to guide patients to an appropriate level of care based on medical need and urgency [1]. This process of guidance is referred to as telephone triage. As medical communication centers often serve as the first point of contact with the health care system for individuals experiencing acute or urgent situations, the quality of the operator’s management of the call is crucial for patient safety [2]. Telephone triage is a knowledge-intensive process, characterized by a multifaceted workflow that relies heavily on human expertise, judgment, and contextual understanding [3,4]. Operators make decisions under time pressure and with limited information while simultaneously using decision support systems, consulting electronic health records, and documenting the calls [5]. This multitasking imposes a considerable cognitive load, potentially reducing attentiveness, efficiency, and communicative ability [6].

Decision support systems are integrated into the operators’ workflow to complement their medical expertise. However, the influence of these systems extends beyond clinical reasoning, as they may also shape the dynamics of the interaction between the operator and the caller [7]. Both the interaction and the caller’s perception of the operator’s competence have been found to be associated with caller satisfaction [8,9]. Moreover, low communication quality has been observed in calls that were inaccurately triaged [10,11]. Together, these findings underscore the importance of ensuring both high medical and communication quality in telephone triage. However, the interaction between the operator and the caller is susceptible to a range of external factors [12], and decision support systems with rigid algorithmic flowcharts and decision trees may constrain the conversation [13-15]. Such systems may therefore be poorly suited to support the flexible and adaptive communication required for telephone triage [3,16].

To improve safety and efficiency in health care, artificial intelligence (AI) is increasingly being integrated into clinical decision-making, including in decision support for emergency medical calls [16-18]. In emergency medical services, clinical applications of machine learning algorithms have so far primarily involved triage and diagnostic classification of individual medical conditions, such as cardiac arrest, cardiovascular disease, or trauma [19]. Designing decision support systems that cover the whole spectrum of conditions encountered in telephone triage is complex due to the diversity of reasons for contact, clinical presentations, and contextual factors.

Recommender systems provide personalized and context‑aware suggestions by analyzing patterns in user behavior. In daily life, most people encounter recommender systems through tailored advertising or suggestions for TV series or movies. In the health care context, recommender systems are often used to influence and motivate individuals to adopt more health-promoting habits [20,21], for example, by providing personalized health feedback via a smartphone app or email [22,23]. Recommender systems are also used to support clinical decision‑making, but there is still a lack of studies examining how such systems influence the actual performance of clinicians [24]. As recommender systems have shown promise in supporting decision‑making by nudging individuals toward better choices without undermining autonomy [25], there may be considerable potential for supporting telephone triage operators through tailored question recommendations that preserve both flexibility and the necessary degree of adaptivity.

Documentation during a call is shaped by whether the operators work with free‑text entries or are supported by templates integrated into the workflow [26]. The documentation process has been described as a situated, personal, and cognitive practice that supports thinking and reflection [27]. Still, writing documentation can be cognitively demanding, as operators must take notes, revise them, and continuously impose structure during the conversation with the callers. Introducing a certain degree of structure can support documentation quality [28], and operators report that templates can provide adaptive structure and serve as a memory aid [29]. It is likely that operators may benefit from a more automatic and structured process of documenting that is displayed in a way that helps them monitor what has been covered so far and identify missing information.

We built a prototype that combined AI-based question recommendations with structured documentation and conducted a feasibility study to test its influence on operators’ performance in a simulated telephone triage setting. We hypothesized that the prototype would affect medical quality without affecting communication quality.

## Methods

### Study Design

This feasibility study used a quasi-experimental pre- and posttest design in which trained simulated callers conducted standardized calls to operators in medical communication centers before and during the use of the prototype. The primary outcomes were overall medical quality and overall communication quality. Secondary outcomes for medical quality included performance on specific medical content items, accuracy of triage, and patient safety, whereas secondary outcomes for communication quality included performance on specific communication items, call duration, and efficiency.

### Study Setting and Sample

In Norway, primary and specialist health care are legally required to provide 24/7 emergency medical services [30]. Emergency primary care, run by the municipalities, is responsible for the population’s access to emergency primary care clinics and local emergency medical communication centers (LEMCs), while specialist health care, run by the state, is responsible for the ambulance service and the ambulance dispatch centers [31]. Through the European number 116117, the LEMCs serve as a gateway to acute and urgent health care by prioritizing patient situations by urgency, initiating responses, and providing medical advice [30]. The LEMCs handle medical calls covering contact reasons ranging from emergencies to nonurgent medical issues, as opposed to ambulance dispatch centers that primarily handle medical emergencies.

The study was conducted at 5 LEMCs in Norway, all of which volunteered to participate after the study objectives had been presented at a national conference for managers in emergency primary care services in March 2022. Each LEMC independently recruited 5 operators. To enable before-after comparisons, the same operators were required to take part in both simulation rounds. Therefore, they had to be fully informed about the study requirements and motivated to complete both phases. To support operator recruitment, an informational video describing the study was shown during staff meetings or distributed via email. Operators willing to participate reported their interest to their manager or a designated contact person at the emergency primary care clinic.

For nonprofessional reasons, 3 operators opted out of the posttest and were therefore excluded from the study. The 22 remaining operators were female registered nurses aged 27-58 years. Among them, 10 operators had 5 years or less of work experience in an LEMC, while 12 had more than 5 years of experience.

The simulated callers were 4 women and 1 man with limited acting experience. They were recruited and trained by an actress who specialized in training simulated patients [32]. One of the simulants withdrew after the pretest and was replaced by a new participant of the same gender and age. Training included 1 individual and 2 group sessions, focusing on the character and context of each case. To ensure medical accuracy, 2 researchers (SLSF and IHJ) with experience in telephone triage served as operators during the training sessions.

Fifteen medical cases representing a range of conditions were developed by the project team. An overview of the cases is provided in Multimedia Appendix 1. Each case described the patient’s condition, contextual details, and specific characteristics of the caller’s role. Without being a fixed script, the case description specified the opening lines that should be provided by the caller at the beginning of the call and information that could be provided later in response to the operator’s questions.

### The Prototype

The prototype was developed as part of the research and innovation project “RE-AIMED: Readjusted Responses by Use of AI in Medical Calls.” Several operators from various LEMCs were involved in the development process. This process is described in detail by Berge et al [5].

**Figure 1:**
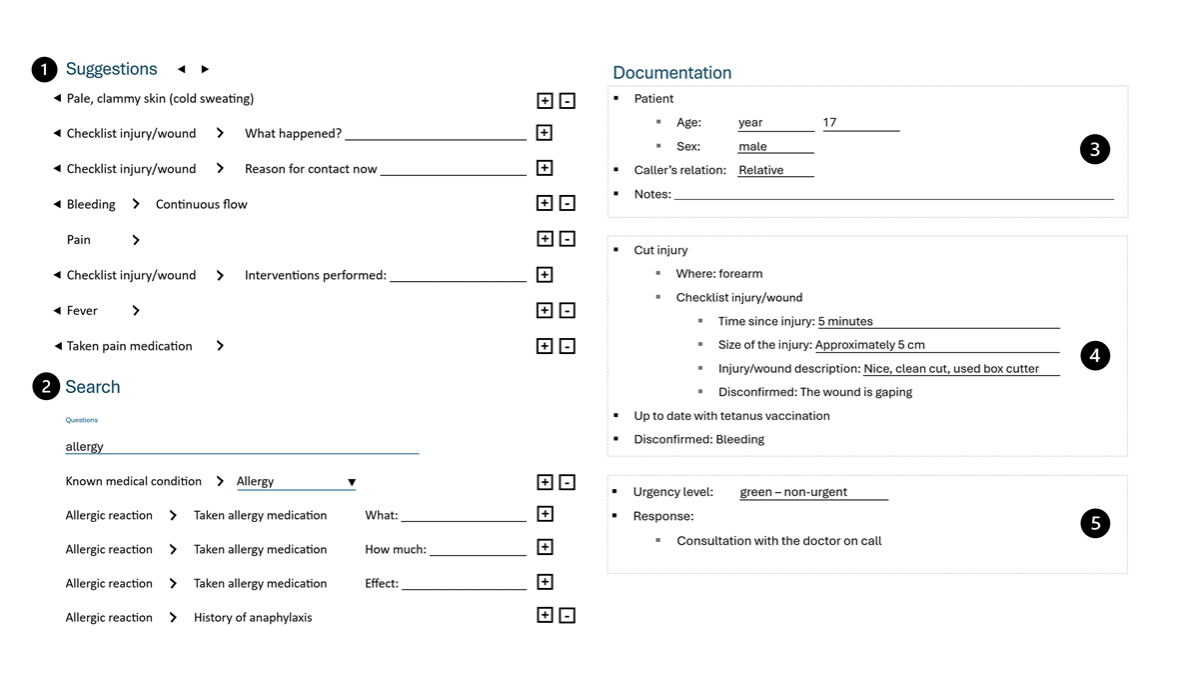
The user interface of the prototype where artificial intelligence–based question recommendations were combined with structured documentation. The numbered labels correspond to numbered sections in the description of the prototype in the text.

The prototype’s user interface contained five different components (Figure 1):

1. Next question prediction: This component presented a dynamically updated list of 8 questions generated using the following machine learning methods: question 1 for identifying highly urgent cases, predicted using classification and regression trees trained on question combinations specific to critical cases; questions 2-4 expanding on topics already introduced in the conversation, predicted using alternating least squares (ALS); questions 5-7 from similar calls, predicted using a combination of cosine similarity and a recurrent neural network (RNN); and question 8 from frequently asked questions not featured higher in the list (no prediction applied). The ALS and RNN models were trained on data from question sequences from registered calls and some synthetic data. The questions were mostly binary and selected from a pool of 1200 predefined questions developed by the project group and refined based on operator feedback.
2. Search field: Option to search for specific questions in the question database.
3. Documentation of patient ID: Fixed fields used to document the patient’s age, sex, the caller’s relationship to the patient, and a free-text field where the operators could take notes to support memory during the call.
4. Documentation of the medical situation: The operator could choose which questions to engage with and document by confirming or negating it. The entries automatically appeared in the documentation and were structured according to a predefined hierarchy. To highlight critical symptom combinations, predicted questions or confirmed symptoms in nearly complete critical combinations were marked with a pink square. If all symptoms in a critical symptom combination were confirmed, the color changed to red. By clicking a marked symptom, operators could see which symptom combinations that would form a high-urgency constellation.
5. Documentation of urgency and response: The operator could choose urgency and response from a drop-down menu.

After the pretest, all operators were introduced to the prototype. They had the opportunity to use it from mid-November 2022 until the completion of the posttest in mid-March 2023. Throughout this period, the operators tested the prototype on anonymous conversations recalled from their own practice. These inputs were used as additional training data for the ALS and RNN models. The operators provided feedback on errors and suggested improvements to the prototype. Follow-up meetings, either digitally or in person, were arranged. Before the posttest, operators used the prototype between 2 and 191 sessions, with a mean of 51 sessions. To support case-relevant question suggestions during the posttest, the recommender system was additionally trained on the 15 simulated cases.

### Data Collection

#### The Intervention

To examine the impact of the prototype on operator performance, a standardized simulation-based protocol was implemented across 2 periods: a pretest in October 2022 and a posttest in March 2023. Each test period lasted 1 week. Each day, 1 operator from each LEMC took part in the simulation. All operators conducted the simulation in their usual work environment while being relieved from regular duties. The simulation content and cases were identical across both periods except for the introduction of the prototype in the posttest. A dedicated group of the authors administered the intervention by providing access to the prototype and overseeing the simulation procedures.

The operators received a checklist with detailed instructions for how the simulation was to be conducted. They were instructed to handle each simulated call as they would in a real‑world setting while also following predefined constraints intended to ensure consistency across operators. They could not consult external resources such as ambulance dispatch centers or on‑call physicians. To preserve ecological realism, operators were given standard guidance on how to handle limitations inherent to simulated callers (eg, reduced geographical familiarity compared with actual residents). The operators were also explicitly instructed not to discuss cases with other participants, in order to avoid influencing each other.

A local telephone number from each LEMC was used so that the operators would recognize the incoming calls as part of the study. Both simulated callers and operators received a schedule specifying the timing of each call. Each scenario was allocated a maximum of 15 minutes for completion. Callers were instructed to place their call within a 10‑minute window. If they were unable to reach the operator within this window, they were instructed to hang up and proceed to the next scheduled call. Any instances of unsuccessful call attempts would be addressed during the longer break periods or at the end of the day. A dedicated member of the author group was available for questions during the simulation weeks.

During the pretest, the operators used their standard equipment, including the integrated communication control system, the electronic health record, and existing decision support tools. During the posttest, operators were instructed to use the prototype to support information gathering and documentation during the calls. The prototype functioned as an add‑on to the existing workflow and was accessed through a standard web browser, where operators logged in using a study‑specific operator ID and password. No incentives were used. Compliance was supported through the scheduled simulation times, relief from regular duties, and detailed procedural instructions.

All calls were audio‑recorded, and all operator documentation was collected at the end of each test week. Files were anonymized and renamed to blind the research team to the test period, location, and operator identity.

#### Assessment of Triage Quality

Calls were evaluated using the validated tool Assessment of Quality in Telephone Triage (AQTT) [33]. AQTT was originally developed in Denmark to evaluate the quality of key aspects in telephone triage calls. Because Danish and Norwegian are linguistically similar, the tool was readily translated into Norwegian. Any uncertainties regarding meaning were clarified through discussions within the author group. AQTT consists of 24 items: 4 overall quality items, 11 items covering medical content, and 9 communication items (Multimedia Appendix 2). Description of the 5-point Likert scale and 7-point triage accuracy scale used are provided in Table 1.

**Table 1:** Description of the 5-point Likert scale and 7-point triage accuracy scale used in the rating tool Assessment of Quality in Telephone Triage.

Scales			Description
**5-point Likert scale**			
	Not applicable	Used only if this aspect was correctly left out.	
	1: Incorrectly omitted	Should have been considered but was incorrectly omitted, and this could potentially have implications for patient safety or serious negative consequences for the patient’s situation.	
	2: Insufficient	Was insufficiently performed. Could potentially have negative consequences for the patient’s situation.	
	3: Sufficient	Was just sufficiently performed. Did probably not have negative consequences for the patient’s situation.	
	4: Good	Was well performed, although there was still room for minor improvements.	
	5: Optimal	Was optimally performed, with no possibility for improvement.	
**7-point triage accuracy scale**			
	1: Severe undertriage	The assigned response level posed a risk of serious consequences for the patient.	
	2: Moderate undertriage	Severe consequences were unlikely, but the assigned priority level was still too low.	
	3: Mild undertriage	The situation could reasonably have been assigned a somewhat higher priority.	
	4: Optimal triage	The decision is considered correct and the most appropriate.	
	5: Mild overtriage	The situation could reasonably have been assigned a somewhat lower priority.	
	6: Moderate overtriage	A less resource-intensive service would likely have been adequate for the situation.	
	7: Severe overtriage	The chosen response level appeared clearly inappropriate and represented a misuse of resources.	

The study applied 23 of the 24 AQTT items:

1. Overall quality (4 items), which were scored based on the rater’s overall perception of the call and rated on a 10-point scale (0 = “very low quality” to 10 = “optimal quality”). The quality items were as follows: (1) communication quality, which covers clear language, effective questioning, structured dialogue, summarizing, and attentiveness to the patient; (2) medical quality, which addresses recognition and prioritization of symptoms, with delivery of relevant medical information; (3) patient safety, which concerns appropriate assessment, provision of a safety net, and reliable advice; and (4) efficiency, which includes timely completion, logical structure, and demonstration of control and overview.
2. Medical content (9 items), of which 8 were scored using a 5-point Likert scale, with an additional “not applicable” (NA) category for cases where an item was correctly left out or available information was insufficient for scoring. Triage accuracy, measured by the item “Selects optimal triage decision,” was rated on a 7-point scale that differentiates between varying degrees of undertriage, which may compromise patient safety, and overtriage, which may lead to unnecessary use of resources. Assessment of triage accuracy was based on the content of the conversation and the decisions made by the operator.
3. Communication (9 items), which were scored using a 5-point Likert scale, with an additional “NA” category for cases where an item was correctly left out or available information was insufficient for scoring.

The 24th item, which was omitted from this study, was item 5 (“Identifies and states the purpose of the patient's call”) from medical content. The item was left out of the study because the simulated callers had a predefined opening line that included a statement of the purpose of the call.

To get familiar with the instrument, 2 authors (SLSF and VM) independently listened to and rated 20 calls before reviewing and discussing the scores. The first author evaluated the remaining calls, and any uncertainties about how to score individual AQTT items or calls were discussed with a dedicated subgroup of the authors. The calls were evaluated in a random order, blinding the rater to whether the call was from the pretest or posttest simulation round.

### Variables

The primary outcomes were overall medical quality and overall communication quality, both measured as continuous variables on a 0-10 scale. Secondary outcomes related to medical quality were assessed using (1) medical content items: 9 categorical items with 5 response categories, (2) triage accuracy: a categorical variable with 7 levels, and (3) patient safety: measured on a continuous 0-10 scale. Secondary outcomes related to communication quality were assessed using (1) communication items: 9 categorical items with 5 response categories, (2) call duration: measured in seconds (continuous variable), and (3) efficiency: measured on a continuous 0-10 scale.

### Data Analysis

The unit of analysis was the individual call. Linear mixed‑effects models were used to compare pretest and posttest scores for the continuous outcomes. Operator and case identifiers were included as random intercepts to account for clustering at both levels. Variance components were used to calculate intraclass correlation coefficients (ICCs) for operators and cases, which were interpreted according to established guidelines [34]. Results are presented with means, mean differences, 95% CIs, corresponding *P* values, and ICCs. Effect sizes for paired comparisons (Cohen *dz*) were calculated by dividing the mean of the paired differences by the standard deviation of those differences [35]. Effect sizes were interpreted according to conventional benchmarks, with values around 0.2 considered small, 0.5 medium, and 0.8 large.

For categorical outcomes, changes between pretest and posttest scores were illustrated descriptively using 100% stacked bar charts. An exception was triage accuracy, which was presented in a 7×7 transition matrix with frequencies. Statistical changes in categorical outcomes were analyzed using Bowker test for table symmetry, with results reported as *P* values. The score category “NA” was used when the item was correctly omitted in a call. Since this omission represented correct performance, this value was recoded to the item’s highest scoring category. A sensitivity analysis was conducted by repeating Bowker test with NA items excluded. This allowed us to assess whether the findings were robust to alternative treatments of NA responses. Statistically significant categorical results were visualized using bubble plots, where bubble size represents the number of observations and the position reflects transitions from pre‑ to posttest scores. A significance level of α=.05 was used for primary outcomes. To account for multiple comparisons, a Bonferroni‑adjusted significance threshold of α=.002 was applied to the secondary outcomes. All analyses were performed using Stata/SE (version 19; StataCorp) [36].

### Ethical Considerations

The study did not fall under the Norwegian Health Research Act, and the Regional Committee for Medical and Health Research Ethics waived the requirement for ethical approval (reference number 227587). The Norwegian Social Science Data Services (reference number 133364) and the Data Protection Officer for Research at NORCE Research AS approved the processing of personal data, including audio recordings and operator documentation. All participants, including operators and the simulated callers, provided written informed consent for participation and for the use of their data for research purposes. Study data were anonymized before analysis, and procedures for handling, storing, and securing data adhered to General Data Protection Regulation and national data protection regulations. Only authorized members of the research team had access to identifiable information. The paper contains no images or supplementary materials that could identify individual participants. Operators received their regular salary from their respective LEMCs for the days they participated in the simulation, while the LEMCs were compensated with NOK 1500 (US $158) per operator per day. Simulated callers were compensated through the performing arts company that provided their services.

## Results

### Paired Calls Included

**Figure 2:**
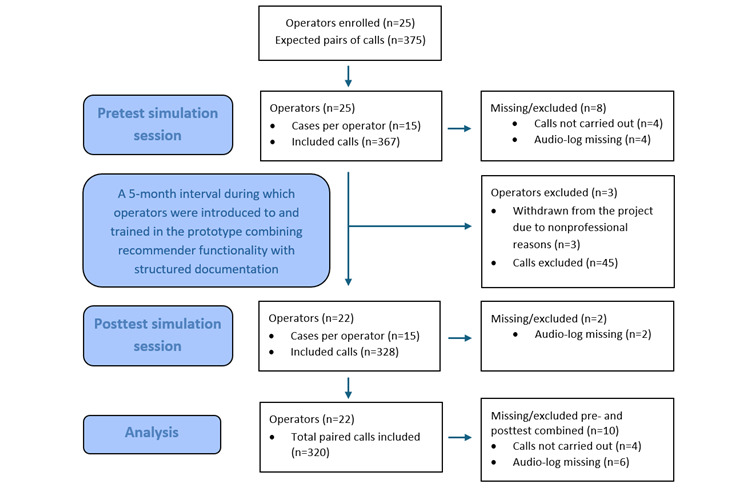
CONSORT (Consolidated Standards of Reporting Trails)–style flow diagram illustrating participant progression through the pretest and posttest simulation sessions, the 5‑month interval including introduction to and training in the prototype combining recommender functionality with structured documentation, reasons for exclusion, and the final number of observation pairs included in the analysis.

Of 330 planned paired calls, 10 were excluded due to unavailability of the caller or the operator at the scheduled call time (n=4) or failure to retrieve the audio recordings (n=6). Thus, 320 paired calls remained for analysis. Participant flow through the pretest and posttest simulation sessions, including exclusions and the final number of paired calls analyzed, is shown in Figure 2.

### Overall Medical and Communication Quality

Overall medical quality improved significantly (difference 0.34, 95% CI 0.11-0.57) (Table 2). Variance components estimated showed an ICC of 0.17 for operators and 0.42 for cases. The effect size was small (Cohen *dz*=0.16). No statistically significant difference was observed in overall communication quality between pretest and posttest (Table 2). The corresponding effect size was very small (Cohen *dz*=0.05).

**Table 2:** Comparison of overall medical and communication quality scores rated on a 10‑point scale, using the Assessment of Quality in Telephone Triage tool in a pre-post feasibility study of 320 paired simulated telephone triage calls handled by operators at local emergency medical communication centers in Norway.

	Pretest, mean	Posttest, mean	Difference	95% CI	ICC^b^ (operator)	ICC (case)	*P* value
Medical quality	*6.83*	*7.16*	*0.34*	*0.11* to *0.57*	*0.17*	*0.42*	.*004*
Communication quality	7.06	6.97	–0.09	–0.28 to 0.10	0.33	0.36	.35

^a^Values in italics indicate statistical significance (*P*<.05).

^b^ICC: intraclass correlation coefficient.

### Secondary Outcomes Related to Medical Quality

**Figure 3:**
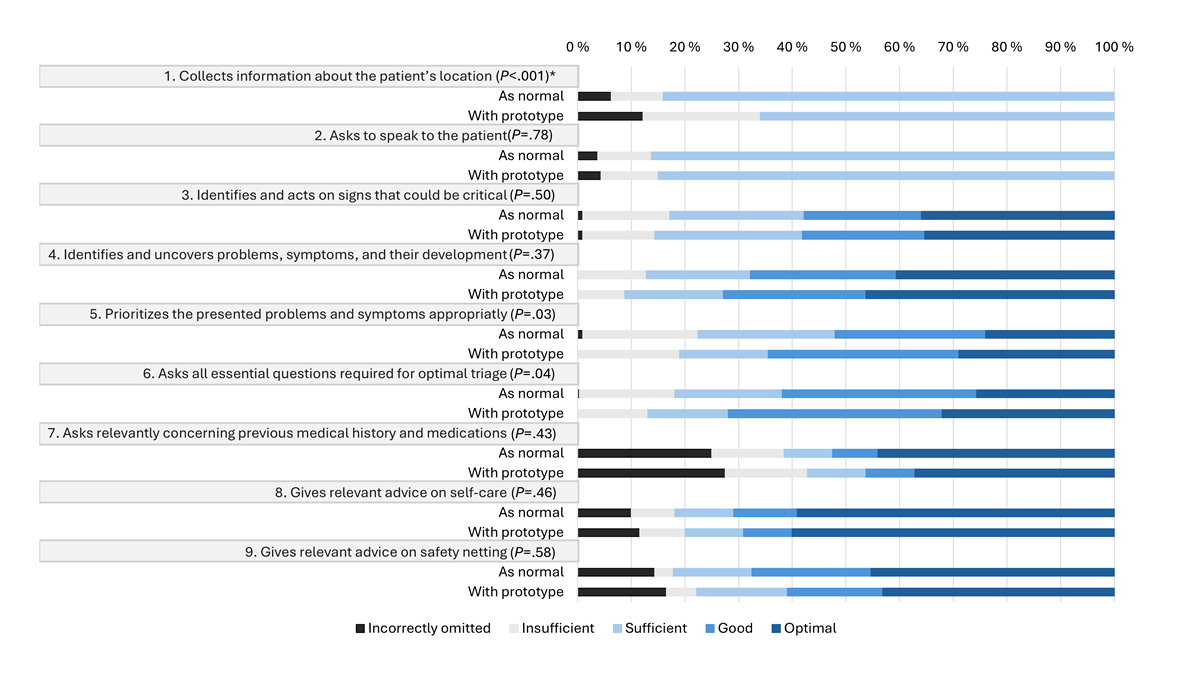
Distribution of scores on medical content items from the Assessment of Quality in Telephone Triage (AQTT) tool in a pre-post feasibility study of 320 paired simulated telephone triage calls handled by operators at local emergency medical communication centers in Norway. The figure compares pretest assessments, in which operators handled calls under usual practice conditions (as normal), with posttest assessments conducted while using a prototype that combined artificial intelligence–based question recommendations with structured documentation (with prototype). It displays the percentage distribution of ratings across the AQTT scoring categories, ranging from “incorrectly omitted” to “optimal,” and includes the *P* value from Bowker test of symmetry to evaluate differences between the pretest and posttest distributions. *Statistical significance (*P*<.002).

Figure 3 illustrates how the scores within each specific medical item were distributed between pre- and posttest. In 16%-43% of the calls, specific items were incorrectly omitted or insufficiently managed.

**Figure 4:**
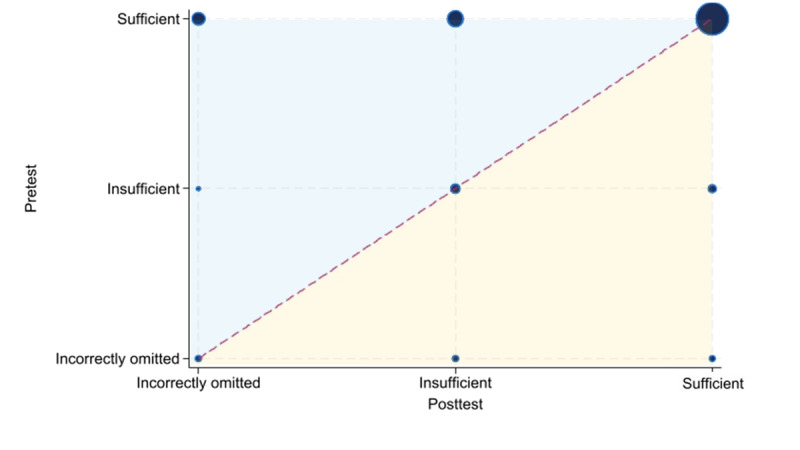
Bubble chart illustrating the agreement between pretest and posttest ratings for the Assessment of Quality in Telephone Triage item “Collects information about the patient’s location” in a feasibility study of simulated telephone triage calls handled by operators at local emergency medical communication centers in Norway. The figure compares pretest assessments, in which operators handled calls under usual practice conditions, with posttest assessments conducted while using the prototype that combined artificial intelligence–based question recommendations with structured documentation. Bubble size represents the number of observations, and bubble position indicates the direction and magnitude of change in performance, with the blue area reflecting higher pretest ratings and the yellow area reflecting higher posttest ratings. The item showed a statistically significant shift in distribution (*P*<.001).

Paired comparisons showed a statistically significant decrease for the item “Collects information about the patient’s location” (*P*<.001) (Figure 4). Otherwise, no statistically significant differences were observed. The sensitivity analysis yielded *P* values comparable with the main analysis (Multimedia Appendix 3), indicating that the results were robust to the treatment of NA responses.

For triage accuracy, 69% (222/320) of cases were optimally triaged in the pretest and 75% (240/320) in the posttest (Table 3). Triage accuracy did not differ significantly between pre-and posttest (*P*=.30). There was also no change between pre- and posttest in overall patient safety, with a mean score of 7.92 in the pretest and 7.99 in the posttest (difference 0.07, 95% CI –0.14 to 0.27; *P*=.53). ICC was 0.14 for operators and 0.41 for cases, and the effect size was small (Cohen *dz*=0.04).

**Table 3:** Pre‑ and posttest assessments of triage accuracy of 320 paired calls in a feasibility study of simulated telephone triage calls handled by operators at local emergency medical communication centers in Norway^a^.

		Posttest							
		**Undertriage**			**Optimal triage**	**Overtriage**			**Total**
**Pretest**		Severe	Moderate	Mild		Mild	Moderate	Severe	
**Undertriage**									
	Severe	0	0	0	0	0	0	0	**0**
	Moderate	0	0	1	5	0	0	0	**6**
	Mild	0	1	15	24	2	1	0	**43**
								
**Optimal triage**		0	2	10	189	16	5	0	**222**
								
**Overtriage**									
	Mild	0	0	1	17	10	2	0	**30**
	Moderate	0	0	0	5	5	7	0	**17**
	Severe	0	0	0	0	0	1	1	**2**
**Total**		**0**	**3**	**27**	**240**	**33**	**16**	**1**	**320**

^a^The table compares pretest assessments, in which operators handled calls under usual practice conditions, with posttest assessments conducted while using a prototype that combined artificial intelligence–based question recommendations with structured documentation. The table differentiates between varying degrees of undertriage, which may compromise patient safety, and overtriage, which may lead to unnecessary use of resources, thereby enabling a comparison of accuracy patterns before and after implementation of the prototype.

### Secondary Outcomes Related to Communication Quality

**Figure 5:**
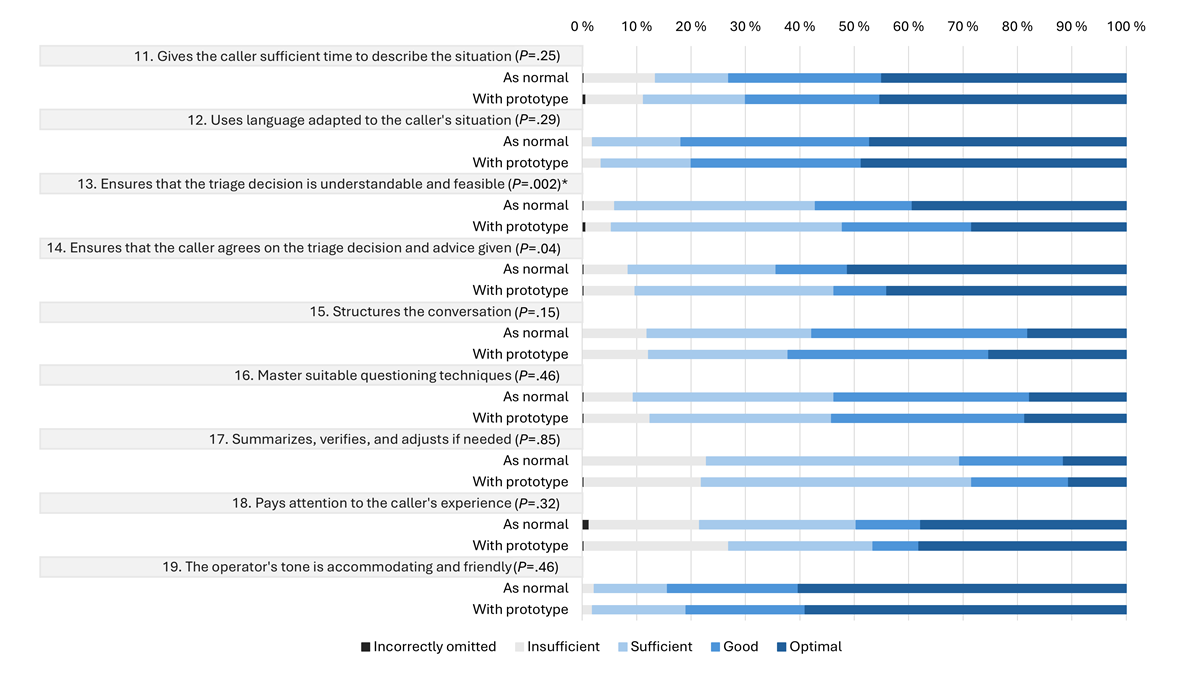
Distribution of scores on communication items from the Assessment of Quality in Telephone Triage (AQTT) tool in a pre-post feasibility study of simulated telephone triage calls handled by operators at local emergency medical communication centers in Norway. The figure compares pretest assessments, in which operators handled calls as usual (as normal), with posttest assessments conducted while using the prototype that combined artificial intelligence–based question recommendations and structured documentation. It displays the percentage distribution of ratings across the AQTT scoring categories, ranging from “incorrectly omitted” to “optimal,” and includes the *P* value from Bowker test of symmetry to evaluate differences between the pretest and posttest distributions. *Statistical significance (*P*<.002).

Figure 5 illustrates how the scores within each specific communication item were distributed between pre- and posttest. In 1%-27% of the calls, the items were incorrectly omitted or insufficiently managed.

**Figure 6:**
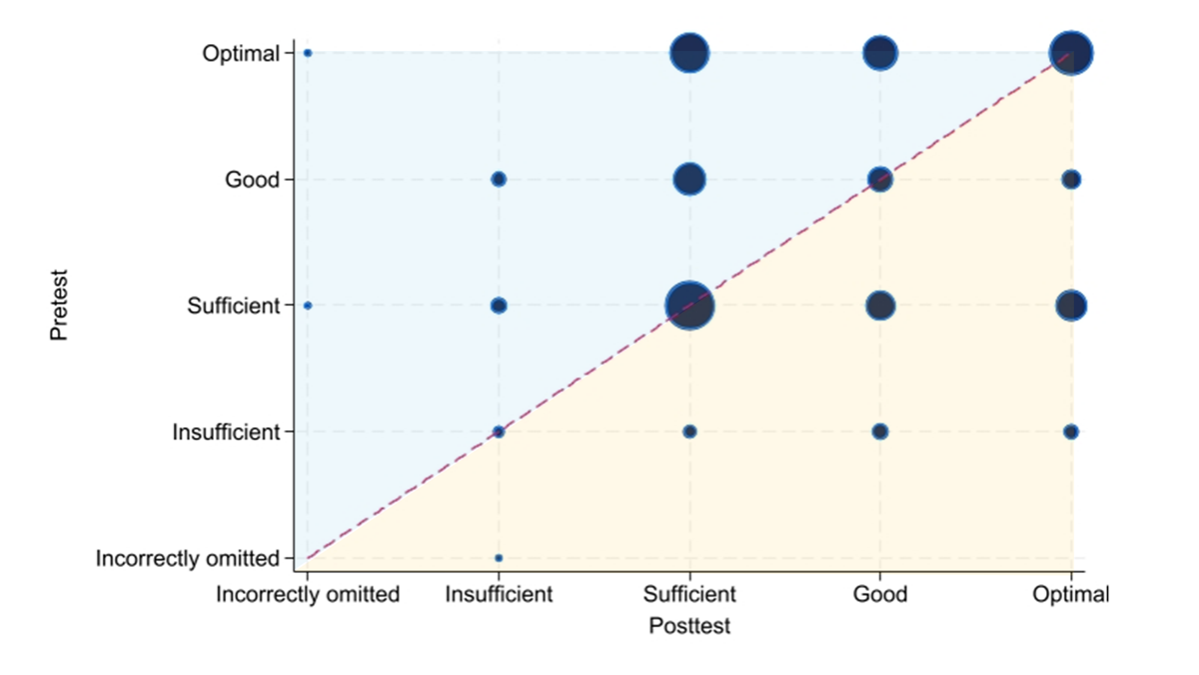
Bubble chart illustrating the agreement between pretest and posttest ratings for the Assessment of Quality in Telephone Triage item “Ensures that the triage decision and the advice given are understandable and feasible” in a feasibility study of simulated telephone triage calls handled by operators at local emergency medical communication centers in Norway. The figure compares pretest assessments, in which operators handled calls under usual practice conditions, with posttest assessments conducted while using the prototype that combined artificial intelligence–based question recommendations with structured documentation. Bubble size represents the number of observations, and bubble position indicates the direction and magnitude of change in performance, with the blue area reflecting higher pretest ratings and the yellow area reflecting higher posttest ratings. The item showed a statistically significant shift in distribution (*P*=.002).

Among the communication items, a statistically significant difference was observed in the item “Ensures that the triage decision and the advice given are understandable and feasible” (*P*=.002), with higher scores in the pretest (Figure 6). Consistent with the sensitivity analysis for the medical content items, the *P* values were comparable with those of the main analysis (Multimedia Appendix 3).

There was no statistically significant change in mean call duration, which was 317 seconds in the pretest and 308 seconds in the posttest (difference –9 seconds, 95% CI –20 to 1 seconds; *P*=.08). ICC was 0.30 for operators and 0.72 for cases, and the effect size was small (Cohen *dz*=0.10). Overall efficiency showed no change from pretest (mean score 6.67) to posttest (mean score 6.84) (difference 0.17, 95% CI –0.08 to 0.41; *P*=.19). ICC was 0.18 for operators and 0.36 for cases, and the effect size was very small (Cohen *dz*=0.07).

## Discussion

### Principal Findings

The results show that use of a prototype, designed to dynamically suggest medical questions and provide structured documentation, influenced the operators’ interactions with the callers in a scenario-limited environment. Overall medical quality improved significantly when the prototype was used, albeit with a small effect size. Secondary outcomes showed a decrease in the items “Collects information about the patient’s location” and “Ensures that the triage decision and the advice given are understandable and feasible.” The remaining overall quality items, specific items, and call duration showed no significant change between the pre- and posttest.

### Comparison With Previous Work

To our knowledge, no previous studies have examined the use of recommender systems during telephone triage. There seems to be a gap in the current evidence base for how health recommender systems lead to behavioral or performance changes, particularly among health professionals [20,21,24,37]. One exception is a study evaluating clinicians’ use of a machine learning–based recommender system during simulated order entry to initiate a component of patient care [38]. Although the recommender did not improve the clinical appropriateness of orders, it was extensively used, perceived as useful by most participants, and associated with a modest increase in ordering behavior. Previous work shows that recommender systems can reduce information overload and provide more targeted, context‑relevant support [21]. One example is the use of AI‑based recommenders in a suicide prevention helpline, which generated real‑time suggestions that were especially used during complex or longer calls [39]. Altogether, this suggests that recommender systems may be useful in health professionals’ work, even when improvements in clinical appropriateness remain limited.

The use of the prototype provided an improvement in overall medical quality, operationalized as recognition and prioritization of symptoms alongside the delivery of relevant medical information. The improvement was not reflected in any of the specific items related to medical content, providing no indication of which aspects of the medical content that changed. In addition, the effect size was small, and no change was observed in triage accuracy or patient safety, indicating that the clinical relevance of the change is limited. It is possible that the prototype’s full potential was not captured in this study as operators already demonstrated high performance during the pretest. This was especially relevant for the overall quality item patient safety, which showed a score of 7.92 out of 10 in the pretest. Such high baseline scores create a ceiling effect, leaving limited room for observable improvement and making small performance changes difficult to detect, even when actual performance may have improved.

The functionalities of the prototype were not tested in isolation, and we cannot determine which specific feature contributed to the observed change. The AI-based question recommender functionality may have contributed to the improvement in medical quality by dynamically presenting context-relevant questions. This may have acted as a subtle nudge, guiding operators toward more comprehensive information gathering. Nudging has been shown in other clinical contexts to influence clinician behavior by drawing attention to relevant cues and thereby enhancing decision‑making [40,41]. In a review, Jesse and Jannach [25] have suggested that a substantial potential of recommender systems lies in their ability to implement digital nudging that supports users in making more informed choices.

Structured documentation has been described by nurses to aid their memory and overview during consultations [29], and conversation‑analytic research demonstrates that structural resources, such as agendas or templates, shape how clinicians organize and navigate interactions [42]. The second functionality of the prototype, structured documentation with high urgency symptom combinations highlighted, dynamically presented the confirmed or denied questions organized according to a predefined hierarchy. This may have helped the operators to keep track of information already covered and to identify remaining or critical information gaps, thus improving the overall medical quality.

Noteworthy, the specific medical item “Collects information about the patient’s location” showed a statistically significant decrease from pretest to posttest. As the instructions given to the operators on how to handle information on the patient’s location was similar before both test rounds, it is unlikely that the decrease can be attributed solely to the simulated setting. The operators may have been influenced by functionalities in the prototype such as questions concerning patient location not being included in the prototype’s question set, and location not being represented within the user interface alongside the standard demographic items (eg, age or sex). Prior research shows that while prompts and nudges can enhance certain behaviors, they may also unintentionally redirect attention away from nonprompted but still essential tasks [43]. This underscores the need to monitor not only the positive effects of such systems but also possible unintended negative consequences for operator performance.

No change in overall communication quality was observed, which is noteworthy given prior concerns that decision support systems might impair the natural communication flow between the operator and the caller [13-15]. Call duration and overall efficiency also remained unchanged. However, when examining specific communication behaviors, fewer operators asked callers to confirm their understanding of the triage decision and advice in the posttest. This suggests that, although the prototype did not reduce communication quality at a global level, it may have subtly shifted the interactional dynamics between operators and callers. Communicative items are typically not embedded in decision support systems but rely on the operators’ adherence to established communication practices [44]. Recent research indicates that AI‑based systems can provide stable and supportive conversational cues, suggesting that integrating communication guidance may enhance both user experience and the thoroughness of information gathering [45]. Given the importance of both accurate information gathering and effective communication for high‑quality telephone triage, future prototypes may benefit from integrating nudges that target both medical content and communicative behaviors.

### Limitations

This study relied on a compound prototype and simulated callers to ensure a controlled comparison between the pretest and posttest conditions, which allowed us to compare the operators’ performance over similar cases in both rounds. However, the study design had several limitations.

First and foremost, the prototype included several components that could influence the operators’ performance. Furthermore, a simulated environment is limited in its ability to capture the variability and unpredictability that characterize real-world calls. These factors limit how the findings can be used in the design of decision support systems for clinical settings.

Second, the recommender system was overfitted by being additionally trained on the very scenarios used in the posttest to generate case‑relevant question suggestions. The amount of relevant training data was limited. By training on the test scenarios, we aimed to test the behavior of a well-performing recommender system. Consequently, the observed improvement cannot be extended to real‑world cases beyond the simulated scenarios.

Third, the use of a single rater introduced a potential risk of researcher bias. To mitigate this, the conversations were assessed using a validated rating tool with dual scoring in the initial phase, and any uncertainties were discussed within a subgroup of the author team throughout the scoring period. Furthermore, calls were scored in random order, and the rater was blinded to the test period, location, and operator identity for each call. Consequently, any remaining rater bias would likely have influenced both test rounds in similar ways.

Fourth, there is a risk of carryover effects because the operators handled the same case twice. Although operators were instructed not to discuss cases with colleagues, and the 2 test rounds were separated by a 5-month interval during which they were estimated to have handled approximately 780-1410 unrelated calls [46], recall bias cannot be ruled out. The long interval and the high volume of intervening calls likely reduced this risk. However, the 5‑month gap also introduces a potential maturation effect, as especially less experienced operators may have naturally improved their professional performance over this period, independent of the intervention.

Fifth, operator recruitment was based on voluntary participation. This may have introduced motivation bias, as more engaged or higher‑performing operators may have been overrepresented among the volunteers. Such a skewed sample may have contributed to the high baseline scores observed, potentially resulting in a ceiling effect and leaving limited room for measurable improvement. The high baseline scores may also be partly explained by the simulated setting, in which the simulated callers used a predefined opening line that stated the purpose of the call. This likely simplified the task compared with clinical reality, where the purpose of the call might be less explicitly stated, potentially inflating overall quality scores.

Sixth, the sample size was set in accordance with the project’s financial, temporal, and personnel constraints, which led to a relatively small sample that may have restricted our ability to conclude. Also, the requirement to use the same operators in both rounds made the study design vulnerable to dropouts. Despite strong motivation to participate, 3 operators withdrew before the second round, reducing an already limited sample available for comparisons and thereby further constraining the ability to detect potential effects of the prototype.

### Future Directions

The findings of this study indicate that a prototype combining recommender functionality with structured documentation can improve medical quality. It seems reasonable to further explore how such functionalities can be developed and incorporated into a full decision support system to ensure both medically accurate support and sustained high‑quality communication during telephone triage. Further studies are needed to understand the contributions of each component of the prototype and to validate the usefulness of question recommenders in settings that have a huge variety in reasons for contact. Operator recruitment should be designed to minimize the risk of ceiling effects by ensuring a more diverse sample of operators, either through broader recruitment or through more randomized selection.

Since the RE-AIMED project started in 2020, advances in AI have accelerated rapidly, with large language models (LLMs) becoming increasingly powerful and accessible. This progress has expanded the use of natural language processing. Compared with traditional models relying solely on structured data, natural language processing–based approaches have been shown to improve classification performance when unstructured free text is incorporated [47]. This opens the possibility for more seamless collaboration between the operator and the machine, where recommendations and documentation are generated directly from the model’s analysis of the ongoing conversation rather than only reacting to the operators’ inputs. Recent work demonstrates that LLMs can provide consistent, context‑sensitive suggestions that enhance both emotional support and information gathering during safety‑related conversations [45], suggesting that LLM‑based systems may likewise assist telephone triage operators by providing real‑time prompts that support empathy, reduce information overload, and improve the overall quality of operator-caller interactions.

### Conclusions

The use of a prototype that combined AI‑based question recommendations with structured documentation yielded a modest improvement in overall medical quality within a scenario‑limited environment. While overall communication quality remained unchanged, aspects of the interaction were negatively affected. It appears feasible that AI‑based question recommendations and structured documentation may serve as useful functionalities within a decision support system. However, these functionalities require further development and evaluation before being used in clinical settings.

## Appendix

Multimedia Appendix 1 Overview of the medical cases presented by the simulated callers, describing the reason for contact, the patient’s sex and age, the caller’s relation to the patient, and the assumed level of urgency.

Multimedia Appendix 2 Overview of the individual items in the assessment tool Assessment of Quality in Telephone Triage (AQTT) and their corresponding rating scales.

Multimedia Appendix 3 Comparison of pretest and posttest values from the evaluation of calls (Assessment of Quality in Telephone Triage items) analyzed by Bowker test for table symmetry (*P* values). The table presents numbers of paired calls in which the item being evaluated was found to be not applicable (NA) in either pretest or posttest, or both tests. NA responses were recoded to the item’s highest scoring category. Sensitivity analysis was performed where NA responses were excluded.

## Abbreviations

AI: artificial intelligence
ALS: alternating least squares
AQTT: Assessment of Quality in Telephone Triage
CONSORT: Consolidated Standards of Reporting Trails
ICC: intraclass correlation coefficient
LEMC: local emergency medical communication centers
LLM: large language model
NA: not applicable
RNN: recurrent neural network

## Data Availability

The data and programming code are not publicly available due to copyright, privacy, and ethical restrictions. The code is stored in a private GitLab repository. The code, reproducibility documentation, simulated caller case descriptions, and the data from this study are available from the corresponding author upon reasonable request.
