# Supplementary material for: Impact of a Prototype Combining Recommender Functionality With Structured Documentation on Operator Performance in Calls to Medical Communication Centers: Quasi-Experimental Feasibility Study
Source: JMIR Form Res. 2026 May 7;10:e87082. doi: 10.2196/87082 (PMC13195374; doi:10.2196/87082)
Supplement: Multimedia Appendix 1 [file formative_v10i1e87082_app1.docx]

| **No.** | **Reason for Contact** | **Patient sex and age** | **Caller** | **Level of urgency** |
| --- | --- | --- | --- | --- |
| 1 | Unconsciousness | Male, 79 | Relative | Acute |
| 2 | Fall | Female, 92 | Relative | Acute |
| 3 | Abdominal pain | Female, 25 | Patient | Urgent |
| 4 | Loss of function | Male, 79 | Relative | Urgent |
| 5 | Heart problems | Male, 47 | Patient | Urgent |
| 6 | Delirium | Female, 85 | Relative | Urgent |
| 7 | Injury at home | Female, 32 | Relative | Urgent |
| 8 | Dizziness | Female, 50 | Patient | Non-urgent |
| 9 | Deliberate self-harm | Male, 17 | Relative | Non-urgent |
| 10 | Bicycle accident | Male, 35 | Patient | Non-urgent |
| 11 | Burn | Female, 59 | Patient | Non-urgent |
| 12 | Cut | Male, 5 | Relative | Non-urgent |
| 13 | Vomiting and diarrhea | Female, 41 | Patient | Non-urgent |
| 14 | Rash | Female, 4 | Relative | Non-urgent |
| 15 | Earache | Female, 2 | Relative | Non-urgent |
