# Supplementary material for: Impact of a Prototype Combining Recommender Functionality With Structured Documentation on Operator Performance in Calls to Medical Communication Centers: Quasi-Experimental Feasibility Study
Source: JMIR Form Res. 2026 May 7;10:e87082. doi: 10.2196/87082 (PMC13195374; doi:10.2196/87082)
Supplement: Multimedia Appendix 2 [file formative_v10i1e87082_app2.docx]

| **Item** | **Descriptions** | **Rating scale** |
| --- | --- | --- |
| ***Medical content:*** | | |
| 1 | Collects information about location | 3-point Likert scale and “not applicable” |
| 2 | Asks to speak to the patient | 3-point Likert scale and “not applicable” |
| 3 | Identifies and acts appropriately on signs that could be critical or life-threatening | 5-point Likert scale and “not applicable” |
| 4 | Identifies and uncovers problems, including symptoms and their development | 5-point Likert scale |
| - | Identifies and states the purpose of the patients call | Excluded |
| 5 | Prioritizes the presented problems and symptoms appropriately | 5-point Likert scale |
| 6 | Asks all essential questions concerning the problems and symptoms to gain the information required for optimal triage | 5-point Likert scale |
| 7 | Asks the relevant questions concerning previous medical history and medications | 5-point Likert scale and “not applicable” |
| 8 | Gives relevant advice on self-care | 5-point Likert scale and “not applicable” |
| 9 | Gives relevant advice on safety netting | 5-point Likert scale and “not applicable” |
| 10 | Selects optimal triage decision | 7-point scale |
| ***Communication:*** | | |
| 11 | Gives the caller sufficient time and space to describe the situation | 5-point Likert scale |
| 12 | The conversation is conducted in understandable language adapted to the caller’s situation | 5-point Likert scale |
| 13 | Ensures that the triage decision and the advice given are understandable and feasible | 5-point Likert scale |
| 14 | Ensures that the caller agrees on the triage decision and advice given and is accommodating in case of disagreement | 5-point Likert scale and “not applicable” |
| 15 | Structures the conversation | 5-point Likert scale |
| 16 | Masters suitable questioning techniques, included suitable use of open-ended, closed-ended and non-leading questions. | 5-point Likert scale |
| 17 | Summarizes, verifies and adjusts if needed | 5-point Likert scale and “not applicable” |
| 18 | Pays attention to the caller’s experience and situation | 5-point Likert scale and “not applicable” |
| 19 | Conducts the conversation in an accommodating and friendly tone | 5-point Likert scale |
| ***Overall quality:*** | | |
| 20 | How would you assess the overall quality of the communication? | 10-point visual analogue scale (0 = “very low quality” to 10 = “optimal quality”) |
| 21 | How would you assess the overall quality of the medical content? | 10-point visual analogue scale (0 = “very low quality” to 10 = “optimal quality”) |
| 22 | How would you assess the overall patient safety? | 10-point visual analogue scale (0 = “very low quality” to 10 = “optimal quality”) |
| 23 | How would you assess the overall efficiency? | 10-point visual analogue scale (0 = “very low quality” to 10 = “optimal quality”) |
