# Supplementary material for: Impact of a Prototype Combining Recommender Functionality With Structured Documentation on Operator Performance in Calls to Medical Communication Centers: Quasi-Experimental Feasibility Study
Source: JMIR Form Res. 2026 May 7;10:e87082. doi: 10.2196/87082 (PMC13195374; doi:10.2196/87082)
Supplement: Multimedia Appendix 3 [file formative_v10i1e87082_app3.docx]

|  | **Not Applicable (NA)** | | | | |  | **Comparison of**  **pre- and post-test** | | | | |  |
| --- | --- | --- | --- | --- | --- | --- | --- | --- | --- | --- | --- | --- |
| **AQTT items** | One test | |  | Both tests | |  | NA as  optimal value | |  | NA excluded^1^ | | |
| ***Medical content:*** | n | % |  | n | % |  | N | p-value |  | n | p-value | |
| 1. Collect information about the patient’s location | 72 | 23% |  | 62 | 19% |  | 320 | <.001^†^ |  | 186 | <.001^†^ | |
| 1. Asks to speak to the patient |  |  |  | 257 | 80% |  | 320 | .776 |  | 63 | .776 | |
| 1. Identifies and acts on signs that could be critical |  |  |  | 44 | 14% |  | 320 | .501 |  | 276 | .501 | |
| 1. Identifies and uncovers problems, symptoms and their development |  |  |  |  |  |  | 320 | .372 |  |  |  | |
| 1. Prioritizes the presented problems and symptoms appropriately |  |  |  |  |  |  | 320 | .031 |  |  |  | |
| 1. Asks all essential questions required for optimal triage |  |  |  |  |  |  | 320 | .044 |  |  |  | |
| 1. Asks relevantly concerning previous medical history and medication |  |  |  | 43 | 13% |  | 320 | .434 |  | 277 | .434 | |
| 1. Gives relevant advice on self-care | 33 | 10% |  | 140 | 44% |  | 320 | .455 |  | 147 | .217 | |
| 1. Gives relevant advice on safety netting | 47 | 15% |  | 53 | 17% |  | 320 | .579 |  | 220 | .268 | |
| ***Communication:*** |  |  |  |  |  |  |  |  |  |  |  | |
| 1. Gives the caller sufficient time to describe the situation |  |  |  |  |  |  | 320 | .247 |  |  |  | |
| 1. Uses language adapted to the caller’s situation |  |  |  |  |  |  | 320 | .288 |  |  |  | |
| 1. Ensures that the triage decision is understandable and feasible |  |  |  |  |  |  | 320 | .002^†^ |  |  |  | |
| 1. Ensures that the caller agrees on the triage decision and the advice given | 79 | 25% |  | 50 | 16% |  | 320 | .038 |  | 191 | .140 | |
| 1. Structures the conversation |  |  |  |  |  |  | 320 | .145 |  |  |  | |
| 1. Master suitable questioning techniques |  |  |  |  |  |  | 320 | .460 |  |  |  | |
| 1. Summarizes, verifies and adjusts if needed |  |  |  | 8 | 3% |  | 320 | .847 |  | 312 | .847 | |
| 1. Pay attention to the caller’s experience |  |  |  | 106 | 32% |  | 320 | .324 |  | 214 | .324 | |
| 1. The operator’s tone is accommodating and friendly |  |  |  |  |  |  | 320 | .457 |  |  |  | |

^†^*Best result in the pre-test* 
*^1^Sensitivity analysis*
